# Detecting Pathogen Exposure During the Non-symptomatic Incubation Period Using Physiological Data: Proof of Concept in Non-human Primates

**DOI:** 10.3389/fphys.2021.691074

**Published:** 2021-09-03

**Authors:** Shakti Davis, Lauren Milechin, Tejash Patel, Mark Hernandez, Greg Ciccarelli, Siddharth Samsi, Lisa Hensley, Arthur Goff, John Trefry, Sara Johnston, Bret Purcell, Catherine Cabrera, Jack Fleischman, Albert Reuther, Kajal Claypool, Franco Rossi, Anna Honko, William Pratt, Albert Swiston

**Affiliations:** ^1^Lincoln Laboratory, Massachusetts Institute of Technology, Lexington, MA, United States; ^2^US Army Medical Research Institute of Infectious Diseases, Ft. Detrick, MD, United States

**Keywords:** machine learning, random forest, physiological signals, incubation period, pre-symptomatic, early infection detection, filovirus and viral hemorrhagic fever, non-human primate

## Abstract

**Background and Objectives:** Early warning of bacterial and viral infection, prior to the development of overt clinical symptoms, allows not only for improved patient care and outcomes but also enables faster implementation of public health measures (patient isolation and contact tracing). Our primary objectives in this effort are 3-fold. *First*, we seek to determine the upper limits of early warning detection through physiological measurements. *Second*, we investigate whether the detected physiological response is specific to the pathogen. *Third*, we explore the feasibility of extending early warning detection with wearable devices.

**Research Methods:** For the first objective, we developed a supervised random forest algorithm to detect pathogen exposure in the asymptomatic period prior to overt symptoms (fever). We used high-resolution physiological telemetry data (aortic blood pressure, intrathoracic pressure, electrocardiograms, and core temperature) from non-human primate animal models exposed to two viral pathogens: Ebola and Marburg (*N* = 20). Second, to determine reusability across different pathogens, we evaluated our algorithm against three independent physiological datasets from non-human primate models (*N* = 13) exposed to three different pathogens: Lassa and Nipah viruses and *Y. pestis*. For the third objective, we evaluated performance degradation when the algorithm was restricted to features derived from electrocardiogram (ECG) waveforms to emulate data from a non-invasive wearable device.

**Results:** First, our cross-validated random forest classifier provides a mean early warning of 51 ± 12 h, with an area under the receiver-operating characteristic curve (AUC) of 0.93 ± 0.01. Second, our algorithm achieved comparable performance when applied to datasets from different pathogen exposures – a mean early warning of 51 ± 14 h and AUC of 0.95 ± 0.01. Last, with a degraded feature set derived solely from ECG, we observed minimal degradation – a mean early warning of 46 ± 14 h and AUC of 0.91 ± 0.001.

**Conclusion:** Under controlled experimental conditions, physiological measurements can provide over 2 days of early warning with high AUC. Deviations in physiological signals following exposure to a pathogen are due to the underlying host’s immunological response and are not specific to the pathogen. Pre-symptomatic detection is strong even when features are limited to ECG-derivatives, suggesting that this approach may translate to non-invasive wearable devices.

## Introduction

Early warning of pathogen exposure, prior to the development of overt clinical symptoms, such as fever, has many advantages: earlier patient care increases the probability of a positive prognosis (Stiver, 2003; Bausch et al., 2010; Bociaga-Jasik et al., 2014; Tosh and Sampathkumar, 2014) and faster public health measure deployment, such as patient isolation and contact tracing (Khan et al., 1999; Eichner, 2003; Pandey et al., 2014), which reduces transmission (Fraser et al., 2004). Following pathogen exposure, there exists an incubation phase, where overt clinical symptoms are not yet present (Evans and Kaslow, 1997). This incubation phase can vary from days to years depending on the virus (American Public Health Association, 1995; Lessler et al., 2009) and is reported to be 3–25 days for many hemorrhagic fevers (Bausch et al., 2010; Eichner et al., 2011; Pavlin, 2014; Tosh and Sampathkumar, 2014) and 2–4 days for *Y. pestis* (Kool and Weinstein, 2005). Following this incubation phase, the prodromal period is marked by non-specific symptoms such as fever, rash, loss of appetite, and hypersomnia (Evans and Kaslow, 1997).

**Figure 1:**
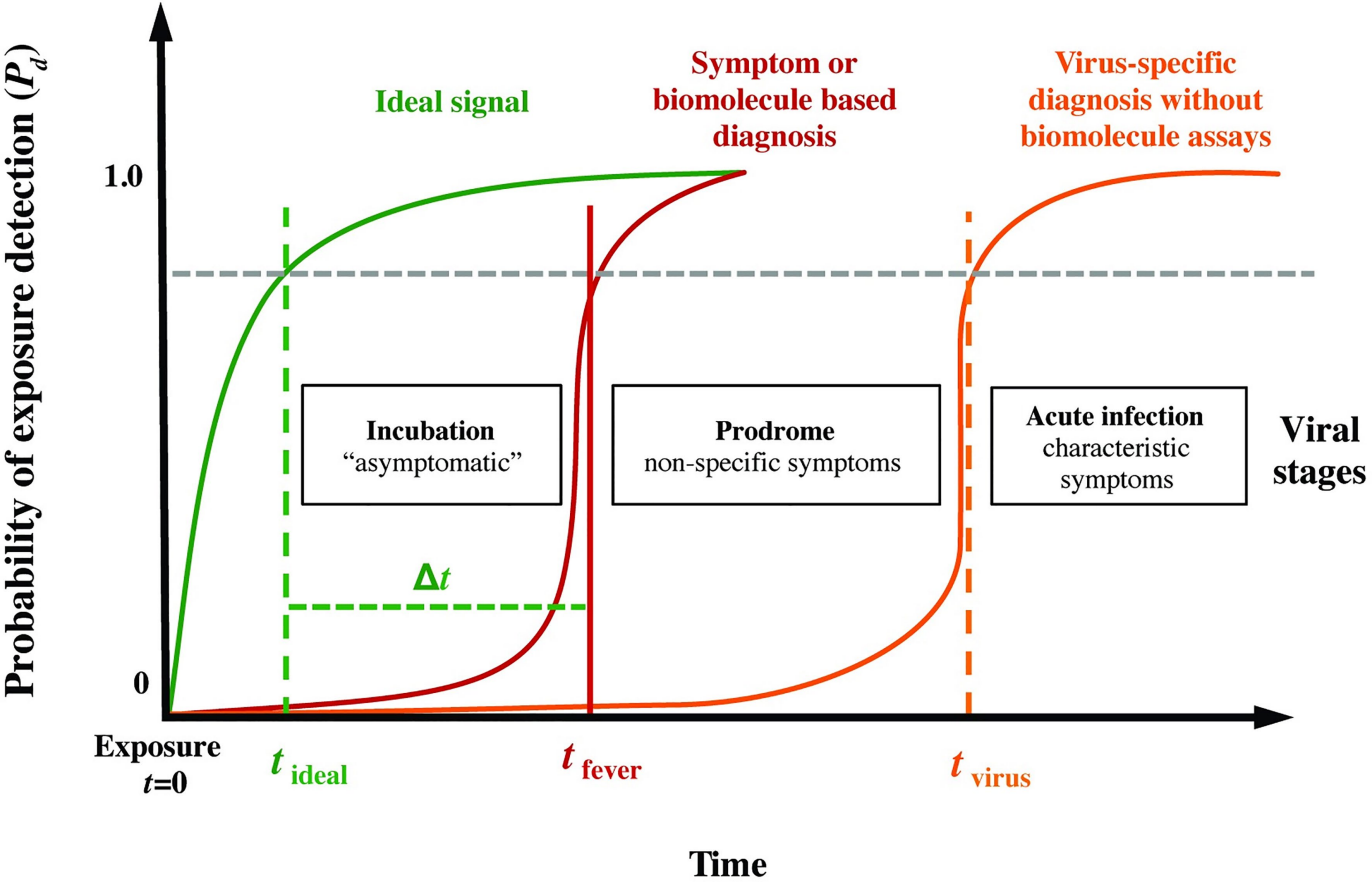
Phases following pathogen exposure. This notional schematic shows the probability of detection (*P_d_*) for current symptoms-based detection (red curve) and an ideal signal (green curve) vs. time (viral exposure at *t* = 0), overlaid with a typical evolution of symptoms. An ideal sensor and analysis system would be capable of detecting exposure for a given *P_d_* (and probability of false alarm, *P_fa_*) soon after exposure and during the incubation period (*t_ideal_*), well before the non-specific symptoms of the prodrome (*t_fever_*). We define the difference *Δt* = *t_fever_* − *t_ideal_* as the *early warning time*.

Figure 1 presents a conceptual model of the probability of infection detection *P_d_* during different post-exposure periods (incubation, prodrome, and virus-specific symptoms) for current specific (i.e., molecular biomarkers) and non-specific (i.e., symptoms-based) diagnostics. Overlaid on this plot, we include an “ideal” sensing system capable of detecting pathogen exposure even during the earliest moments of the incubation period. We hypothesized that quantifiable abnormalities (relative to a personalized baseline, for instance) in high-resolution physiological waveforms, such as electrocardiograms, blood pressure, respiration, and temperature, *before* overt clinical signs could be a basis for the ideal signal in Figure 1, thereby providing advanced notice (the early warning time, *Δt* = *t_fever_* − *t_ideal_*) of imminent pathogen-induced illness.

In addition to characteristic clinical presentations, most infectious disease diagnosis is based upon the identification of pathogen-specific molecular signatures (*via* culture, PCR/RT-PCR or sequencing for DNA or RNA, or immunocapture assays for antigen or antibody) in a relevant biological fluid (Evans and Kaslow, 1997; Ksiazek et al., 1999; Bausch et al., 2000; Drosten et al., 2002, 2003; Mahony, 2008; Muldrew, 2009; Kortepeter et al., 2011; Sedlak and Jerome, 2013; Liu et al., 2014b). Exciting new approaches enabled by high-throughput sequencing have shown the promise of pre-symptomatic detection using genomic (Zaas et al., 2009; Woods et al., 2013) or transcriptional (Malhotra et al., 2013; Caballero et al., 2014; Connor et al., 2015; Speranza et al., 2018) expression profiles in the host (Shurtleff et al., 2015). However, these approaches suffer from often prohibitively steep logistic burdens and associated costs (cold chain storage, equipment requirements, qualified operators, and serial sampling); indeed, most infections presented clinically are never definitively determined etiologically, much less serially sampled. Furthermore, molecular diagnostics are rarely used until patient self-reporting and presentation of overt clinical symptoms such as fever.

Previous work on physiological signal-based early infection detection work has been heavily focused on systemic bacterial infection (Korach et al., 2001; Chen and Kuo, 2007; Ahmad et al., 2009; Papaioannou et al., 2012; Scheff et al., 2012, 2013b), and largely centered upon higher sampling rates of body core temperature (Williamson et al., 2007; Papaioannou et al., 2012), advanced analyses of strongly-confounded signals such as heart rate variability (Korach et al., 2001; Chen and Kuo, 2007; Ahmad et al., 2009) or social dynamics (Madan et al., 2010), or sensor data fusion from already symptomatic (febrile) individuals (Sun et al., 2013). While great progress has been made in developing techniques for physiological-signal based early warning of bacterial infections and other critical illnesses in a hospital setting (Heldt et al., 2006; Liu et al., 2011, 2014a; Lehman et al., 2014), efforts to extend these techniques to viral infections or other communicable pathogens in non-clinical contexts using wearable sensor systems have only recently been pursued in observational studies on human subjects, primarily as a rapid response to the COVID-19 pandemic (Li et al., 2017; Miller et al., 2020; Mishra et al., 2020; Natarajan et al., 2020; Quer et al., 2021). While rapid progress has been made in detecting COVID-19 in humans using physiological signals, the uncontrolled conditions inherent in an observational study preclude the type of systematic analyses that are possible with controlled animal models.

In this paper, we present a proof-of-concept investigation on detecting pathogen exposure from physiological measurement data. We leverage telemetry data collected on animal models, where the exposures are well characterized in terms of the challenge time and the route, dose, and strain of the pathogen exposure. We focus the investigation around three research questions: (1) what are the upper limits for detecting pathogen exposure based solely on physiological measurements? (2) are the indications derived from physiological measurements specific to a pathogen? and (3) is it feasible to use non-invasive wearable sensors to monitor for illness?

## Materials and Methods

**Figure 2:**
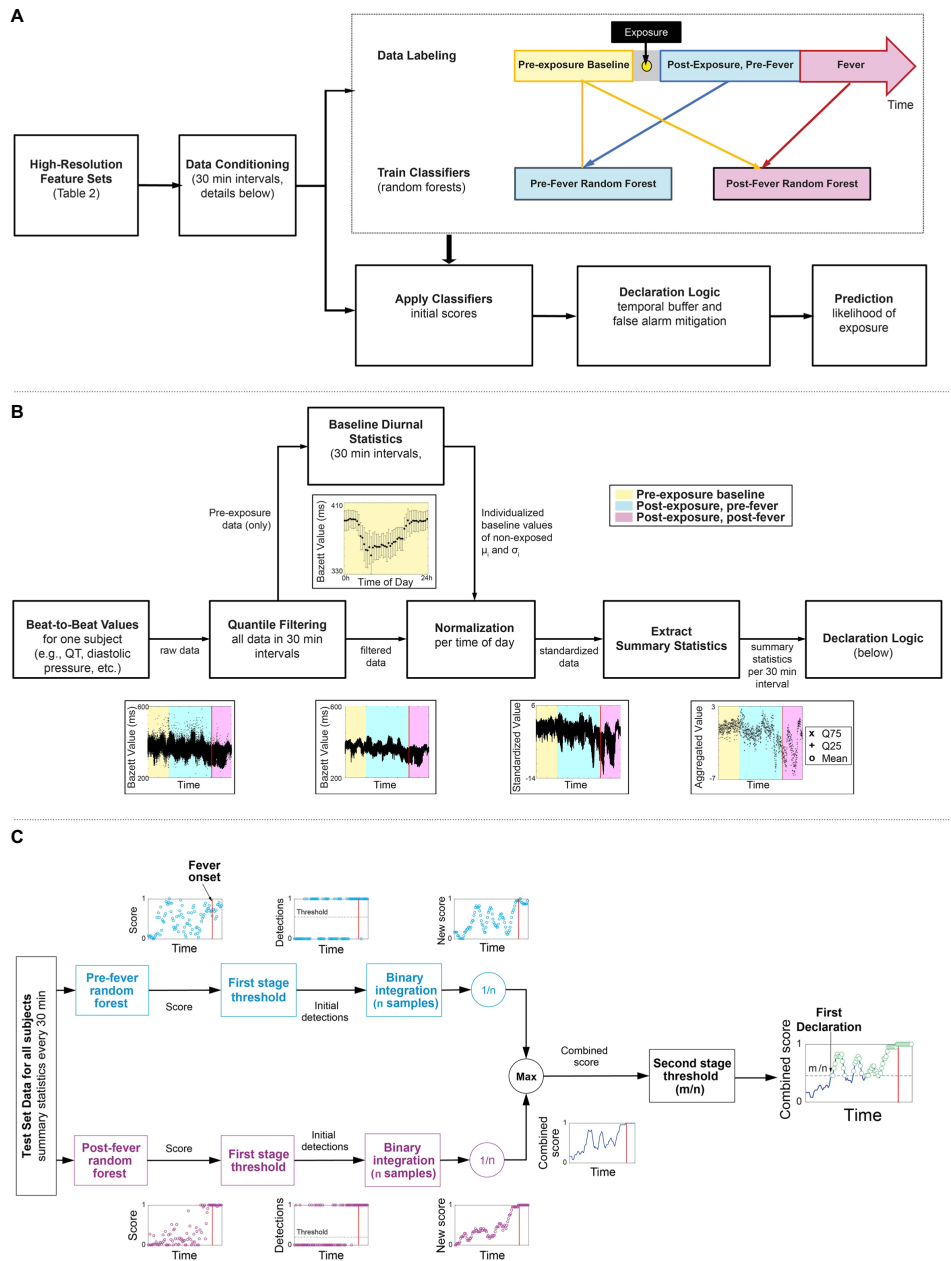
Overview of the workflow of our early warning algorithm. **(A)** Top level approach including the timeline for labeling the data. **(B)** Detail on how beat-by-beat data is conditioned to remove noise and diurnal cycles and summary statistics are extracted as features for the two classifiers. **(C)** A block diagram of a two-stage detection logic to reduce false alarms.

Figure 2A outlines our overall methodology: telemetry systems continuously measured and recorded physiological data for six non-human primate model studies, where the animals were exposed to different viral or bacterial pathogens. The data are labeled and pre-processed to remove time dependence and extract summary features. Using a subset of the studies, we develop two random forest classifiers to detect the signs of pathogen exposure during the pre-fever and post-fever time periods and leverage a binary integration technique to add an element of memory into the model and control the false alarm rate. We describe these, our core methods, together with the fine tuning of our model parameters and performance evaluation of our trained models against the held-out studies here. Using this methodology, we determine the upper limits of sensitivity to detect pathogen exposure that can be achieved in these controlled conditions.

### Data Set: Animal Studies

Table 1 provides a summary of the six non-human primate studies conducted at US Army Medical Research Institute of Infectious Diseases (USAMRIID). All animal studies were conducted under an Institutional Animal Care and Use Committee (IACUC) approved protocol in compliance with the Animal Welfare Act, PHS Policy, and other Federal statutes regulations relating to animals and experiments involving animals; the research facility is accredited by the International Association for Assessment and Accreditation of Laboratory Animal Care and adheres to principles stated in the Guide for the Care and Use of Laboratory Animals, and the National Research Council, 2011.

**Table 1:** Summary of non-human primate studies used.

Study	Pathogen	Exposure method	Species	Total subjects	Exclusions number, reason	Subjects analyzed (M, F)	Days of data mean ± std	Days to fever mean ± std	Telemetry system(s)
1	MARV (Ewers et al., 2016)	Aerosol	Rhesus	5	0	5 (3, 2)	14.4 ± 0.3	4.0 ± 0.4	ITS T27F
2	MARV	IM	Cynomolgus	11	2, premature fever	9 (7, 2)	11.4 ± 0.9	3.9 ± 1.5	ITS T27F
3	EBOV	Aerosol	Cynomolgus	15	1, corrupt signal 8, therapeutic interventions	6 (3, 3)	12.0 ± 2.6	3.9 ± 0.3	ITS T37F; DSI L11
4	NiV (Johnston et al., 2015)	IT	African green monkey	7	2, premature fever	5 (5, 0)	15.6 ± 7.1	4.1 ± 1.8	ITS T27F
5	LASV (Malhotra et al., 2013)	Aerosol	Cynomolgus	4	0	4 (4, 0)	24.2 ± 15.2	3.5 ± 0.4	ITS T27F
6	*Y. pestis*	Aerosol	African green monkey	4	0	4 (4, 0)	10.3 ± 1.2	3.2 ± 1.0	ITS T27F

In studies 1 and 2, rhesus and cynomolgus macaques, respectively, were exposed to the Marburg Angola virus referred to as MARV (Marburg virus/H.sapiens-tc/ANG/2005/Angola-1379c – USAMRIID challenge stock “R17214”). In study 3, cynomolgus macaques were exposed to Ebola virus (EBOV) at a target dose of 100 plaque forming units (pfu; Ebola virus/H.sapiens-tc/COD/1995/Kikwit-9510621; 7U EBOV; USAMRIID challenge stock “R4415;” GenBank # KT762962). In study 4, African green monkeys were exposed to the Malaysian Strain of Nipah virus (NiV) isolated from a patient from the 1998 to 1999 outbreak in Malaysia and provided to USAMRIID by the Centers for Disease Control and Prevention. In study 5, cynomolgus macaques were exposed to the Josiah strain of the Lassa virus (LASV; challenge stock “AIMS 17294;” GenBank #s JN650517.1, JN650518.1). In study 6, African green monkeys were exposed to *Yersenia pestis* (*Y. pestis*), a causative bacterial agent of bubonic and pneumonic plague. Additional details on studies 1, 5, and 6 have been published elsewhere (Malhotra et al., 2013; Johnston et al., 2015; Ewers et al., 2016). Dependent on the study, animals were exposed under sedation *via* either aerosol, intramuscular (IM) injection, or intratracheal (IT) exposure (see Table 1).

In each study, the animals were implanted with remote telemetry devices (Konigsberg Instruments, Inc., T27F or T37F, or Data Sciences International Inc. L11: see details in Table 1) 3–5 months before exposure, and, if used, with a central venous catheter 2–4 weeks before. They were then transferred into BSL-3 (bacterial exposures) or BSL-4 (viral exposures) containment 5–7 days prior to challenge. Baseline data from the telemetry devices were collected for 3–7 days before exposure. Monitoring *via* the telemetry devices continued until death or the completion of the study. The mean duration of recordings within each study is shown in Table 1. The exposure time (*t* = 0) denotes the time of IM injection or IT exposure or when a subject was returned to the cage following aerosol exposure (~20 min).

Data from a total of 46 animal subjects from the six studies were available. Eight subjects from the EBOV exposure study were excluded from post-exposure analysis because they received therapeutic interventions following the challenge, which could be a confounding factor. Five subjects across the cohorts were excluded on the basis of either substantial data loss from equipment failure or development of fever more than 2 days prior to the studies’ mean (i.e., possible co-morbid infections or complications). This resulted in a total of 33 animal subjects for our analytical cohort (*N* = 33).

Multimodal physiological data from the animal subjects were made available in NSS format (Notocord Systems, Croissy-sur-Seine, France). The multimodal physiological data from the implanted telemetry devices included raw waveforms of the aortic blood pressure (sampling frequency *f_s_* = 250 Hz), electrocardiogram (ECG; *f_s_* = 500 Hz), intrathoracic pressure (*f_s_* = 250 Hz), and core temperature (*f_s_* = 50 Hz). All signals were measured internal to the animals, which generally resulted in very high-signal fidelity. Using Notocord software, we extracted the features listed in Table 2 from the raw waveforms.

**Table 2:** List of extracted physiological features and the raw waveform from which they are derived.

Feature name prefix	Raw signal	Description
AOPAMean	Aortic blood pressure	Approximated mean arterial pressure between two successive diastoles, 1/3*P_systolic_ + 2/3*P_diastolic_
AOPDiastolic	Aortic blood pressure	Aortic pressure during diastole
AOPSystolic	Aortic blood pressure	Aortic pressure during systole
RR	Electrocardigram	Interbeat interval measured between adjacent R peaks
HR	Electrocardigram	Heart rate computed between two successive diastoles from the ECG waveform (inverse of RR)
PR	Electrocardigram	Time interval between P and R waves
QRS	Electrocardigram	Time interval between Q and S waves
QT	Electrocardigram	Time interval between Q and T waves
Bazett	Electrocardigram	QT interval corrected per the Bazett method, $QT/\sqrt{RR}$ (Bazett, 1920)
Fridericia	Electrocardigram	QT interval corrected per the Fridericia method, $QT/\sqrt[{3}]{RR}$ (Fridericia, 2003)
RespMean	Intrathoracic pressure	Mean respiratory rate calculated over a non-overlapping 200 s time window
Temp	Temperature	Core temperature

For each feature name prefix, three summary statistics are computed with suffixes _Mean, _Q25, and _Q75 for the mean, 25^th^ and 75^th^ quantiles, respectively.

### Data Labeling

We categorized all features retrieved from the Notocord system (Table 2) as: *pre-exposure baseline* or simply *baseline* – data collected from the start of the recording up to 12 h before the viral or bacterial challenge; and *post-exposure* – data collected 24 h after the viral or bacterial challenge until death or the completion of the study. Relative timing of the labeled regions is depicted in Figure 2A. Data from 12 h before and 24 h after viral or bacterial challenge were excluded from performance metrics due to differences in animal handling and exposure sedation that resulted in significant physiological deviations from baseline data unrelated to pathogen infection.

All subjects across all six studies developed fever as a result of the pathogen exposure. For early warning, fever onset is an important reference point. We define fever onset as the first time that the subject’s core temperature measurement exceeds 1.5°C above that subject’s diurnal baseline (Laupland, 2009) with the additional constraint that the temperature is sustained above threshold for at least 2 h. Leveraging this fever onset, we further categorized the post-exposure data as being *pre-fever* – before onset of fever; or *post-fever* – after onset of fever.

### Data Pre-processing: Conditioning Physiological Data

Figure 2B illustrates the key steps taken to condition the features (Table 2) extracted from the Notocord system after the data labeling. These steps are applied per subject and per feature to reduce diurnal and inter-subject time dependencies in the data. As an example, the time series feature, Bazett, which represents QT-corrected intervals from an ECG waveform are shown at each processing stage.

#### Step 1: Quantile Filtering

We apply quantile filtering to remove any outliers that result due to motion, poor sensor placement, or intermittent transmission drop outs. We batch process the raw beat-to-beat values for the feature/subject pair in non-overlapping intervals, *k*-minutes per epoch, and omit local outliers from the top and bottom 2% of each interval.

#### Step 2: Baseline Diurnal Statistics

We estimate diurnal statistics (mean, *μ_i_*, standard deviation, *σ_i_*) for each *i^th^* interval of a 24 h day across all baseline days. For example, consider a *k* = 60 min epoch and the *i* = 1 interval spanning 12:00 AM to 1:00 AM, then for a single subject, we find all the feature samples from baseline days that were measured within the 1st hour of their respective day. From this multi-day set, we compute *μ*_1_ and *σ*_1_, which represent the subject’s baseline between the hours of 12:00 AM and 1:00 AM. We repeat this for each hour of the day, *i* = 2, 3, …, 24, each feature, and each subject to construct individualized baseline diurnal profiles, as illustrated in Figure 2B.

#### Step 3: Normalization

Using the baseline mean, *μ_i_*, and standard deviation, *σ_i_* for each interval *i*, we normalize all corresponding intervals (*i*) in the pre- and post-exposure data, thereby removing the diurnal time dependence from the data.

#### Step 4: Extract Summary Statistics

Lastly, we down-sample the standardized, high-resolution (beat-to-beat) data obtained from Step 3 by extracting summary statistics from each *l*-minute epoch. The summary statistics include mean, 25^th^ quantile and 75^th^ quantile. Extracting the summary statistics serves to characterize the underlying distribution within an epoch, but also provides time alignment across the different feature sources, which may be sampled at disparate rates. Note that the *k*-minute epoch selected for steps 1–3, and *l*-minute epoch selected for step 4 need not be the same length.

### Random Forest Ensemble

We train our random forest models on two post-exposure stages, thus allowing the algorithms to adapt to different physiological cues during the pre-fever and post-fever phases. The pre-fever random forest model is optimized to discriminate the earliest stages of illness by training it on pre-fever data samples vs. baseline. The post-fever random forest learns discriminants of the febrile phase of illness by training it on post-fever data samples vs. baseline. The number of data points used for training is balanced for equal representation of the classes.

Both models, pre-fever and post-fever, are trained using the *l*-minute epoch summary statistics generated in the data pre-processing step, including mean, 25^th^ and 75^th^ quantiles for all 12 features listed in Table 2.

The models are implemented using the TreeBagger class in the MATLAB Statistics and Machine Learning Toolbox.

### Detection Logic

We next apply a two-stage detection process, depicted in Figure 2C, to the prediction scores generated by the pre- and post-fever models for each *l*-minute epoch, with the primary goals of reducing the overall false alarm rate and incorporating recent historical scores in the decision.

In stage one of the detection process, a time series of feature vectors is processed on two parallel paths. One path calculates a pre-fever random forest score while the other path independently calculates a post-fever random forest score. On each path, the score is compared to a threshold associated with the respective model (threshold selection is described in section “Model Tuning”). Initial detections occur when a score exceeds the threshold. To reduce the likelihood of spurious detections, we buffer the initial detections over a window of *n* epochs and perform binary integration (Shnidman, 1998), calculating a moving average over an l*n minute window.

In stage 2 of the detection process, the parallel paths are reunited by taking the maximum of the pre-fever and post-fever moving average value at each epoch. This combined score is compared to a second stage threshold of *m*/*n*, where *m* is an integer such that *m* ≤ *n*. Combined scores in excess of *m*/*n* are declared to be in the exposed class, and we use the term “declaration” to denote the final decision from the two-stage processing. Note that the buffering aspect of binary integration imposes some latency on the system, so no declarations are reported in the first l*n minutes.

### Performance Metrics

We evaluate overall performance of our models using three key performance metrics: probability of detection, *P_d_*, probability of false alarm, *P_fa_*, area under the receiver operating curve (AUC), and mean early warning time *Δt*.

We calculate the probability of correct declaration, *P_d_*, as the number of true positive declarations over the total number of post-exposure samples. In addition, we compute *P_d_* on the subset of pre-fever and post-fever samples. We use the term, system *P_d_*, to represent correct detection over all post-exposure data samples regardless of fever status, while pre-fever *P_d_* indicates the refinement, where correct detections are evaluated exclusively on the subset of pre-fever data samples. The probability of false alarm, *P_fa_*, (also referred to as the system *P_fa_*) is defined as the number of false positive declarations over the total number of baseline samples. In order to estimate small false alarm rates with meaningful precision, we require a large number of baseline data samples. For false alarm analysis, we supplement with baseline data from some animals that were excluded from the primary analysis. These data include seven full days of measurements from each of nine animals prior to pathogen exposure: seven subjects from the EBOV study (excluded due to therapeutic intervention following exposure) and two subjects from the NiV study (which developed fever earlier than our exclusion criteria). We compute 95% confidence intervals for *P_d_ and P_fa_ assuming normal distribution as the number of trials is large (the number varies depending on the dataset and metric under evaluation, but is greater than 500 for all scenarios considered here)*.

We generate receiver operating characteristic (ROC) curves to measure system performance by calculating *P_d_* vs. *P_fa_* at a series of threshold values (sweeping the first-stage detection threshold while holding the second-stage *m*/*n* threshold constant) and report the AUCs evaluated against pre-fever and post-fever data samples.

Another important measure of system performance is the mean early warning time, *Δt*. The early warning time for an individual subject is defined as the time of the first true declaration minus the time of fever onset. We compute the mean across all subjects to characterize the early warning time afforded by the system and report 95% confidence interval based on a *t*-distribution since the number of subjects is small (<30 for the two subgroups considered here).

### Performance Evaluations

We evaluate detection performance under three distinct scenarios to address our core research questions. First, to answer the fundamental question of how well pathogen exposure can be detected based solely on physiological measurements, we focus on data from the subset of *N* = 20 animal subjects from the EBOV and MARV studies (Studies 1–3). We develop our algorithms using a 3-fold cross-validation approach, which has been shown to perform better (Shao, 1993) than leave-one-out validations for small dataset. This approach explicitly varies five experimental variables (species and sex of animal, exposure route, pathogen, and target dose; see Table 1) across the three partitions, which reduces the likelihood of biasing the model for any particular condition.

Second, to evaluate whether the early warning capabilities of our algorithm extend to other pathogens, we train the models on the *N* = 20 subjects from EBOV and MARV and apply them to an independent dataset of *N* = 13 animal subjects from the LASV, NiV, and *Y. pestis* studies (Studies 4–6).

For the third research question, we investigate performance degradation when the inputs of the classifier are restricted to emulate a limited set of measurements that could be obtained with a non-invasive wearable device. While direct measurement of aortic blood pressure, core temperature, and intrathoracic pressure rely on invasive or intrusive sensors, ECG signals can be readily measured with wearable sensors. For this evaluation, we limit the classifier inputs to the set of EGG-derived features. This scenario is also trained on the EBOV and MARV data set and applied to the LASV, NiV and *Y. pestis* data set.

### Model Tuning

Model tuning, including feature selection and other classifier and detection parameters is performed using systematic parameter sweeps within the subset of *N* = 20 animals exposed to EBOV and MARV. Table 3 summarizes the tunable parameters from sections Data Pre-Processing: Conditioning Physiological Data, Random Forest Ensemble, and Detection Logic.

**Table 3:** Algorithm parameters.

Parameter	Description	Stage of algorithm	Value
*k*	Epoch length	Feature normalization	30 min
*l*	Epoch length	Summary statistics	30 min
*n_trees_*	Number of trees in random forest	Classifier training	15
*n_features_*	Number of features in random forest	Classifier training/testing	10
*Feature sets*	Set of most important features on which the random forest is grown	Classifier training/testing	See Table 4
*t_pre-fever_*	First-stage threshold for the pre-fever random forest scores	Detection logic	0.75
*t_post-fever_*	First-stage threshold for the post-fever random forest scores	Detection logic	0.21
*m*	Second-stage threshold	Detection logic	11
*n*	Binary integration buffer length	Detection logic	24 epochs (12 h)

To characterize performance as a function of different epoch lengths and number of trees, we make use of the random forest out-of-bag errors. Random forest ensembles are generated through an aggregated bagging process, whereby a random subset or “bag” of data points is selected with replacement to build a decision tree. The process is repeated until a specified number of trees are generated. Out-of-bag errors are calculated during training by evaluating decision trees against the samples that were not in their bag, providing a convenient assessment of classifier performance. We sweep the parameter values for *k*, *l*, and *n_trees_* and consider tradeoffs for both the out-of-bag errors and the computation times for pre-processing the data.

For feature selection and determining the number of features, *n_features_*, we assign one of the three cross-validation partitions for parameter tuning and the remaining partitions for model training and performance validation. We use a backward elimination feature selection method, leveraging the out-of-bag errors to iteratively identify and drop the feature ranked least important. The impact of varying *n_feature_* is further characterized by the *P_fa_* and pre-fever *P_d_* in this parameter tuning partition.

First-stage detection thresholds are selected based on a user-defined target *P_fa_*, evaluated at the final stage of the declaration logic. We sweep the first-stage thresholds independently for the pre-fever and post-fever classifier and select the smallest threshold for each model that achieves a target *P_fa_* ≤ 0.01.

For the second-stage detection parameters, we sweep *m* and *n*, from *n* = 1 (30 min) to *n* = 36 (18 h) and *m* = 1, 2,…, *n*. For each pair (*m*, *n*), we evaluate performance in terms of the mean early warning time *Δt* and AUC. We also consider the performance when *m* is set to the estimated optimal threshold for a constant (non-fluctuating) signal in noise, $m_{opt}\approx 10^{-0.02}n^{0.8}$ (Shnidman, 1998).

## Results

We present our results in four parts. Section “Parameter Selection” describes the result of parameter sweeps for model tuning and justifies the parameter values used in the algorithms. The remaining three subsections show the resulting detection performance related to our three research objectives: section “Detection Performance for 20 Subjects Exposed to Ebola or Marburg Virus” demonstrates performance within the cross-validation data set, section “Applicability of Pre- and Post-fever Models to Other Pathogens” evaluates performance when the algorithms are applied to pathogens other than the ones they were trained on, and section “Emulation of Early Warning Performance for Wearable Systems” evaluates performance on a limited feature set that could be measured by wearable devices.

### Parameter Selection

**Figure 3:**
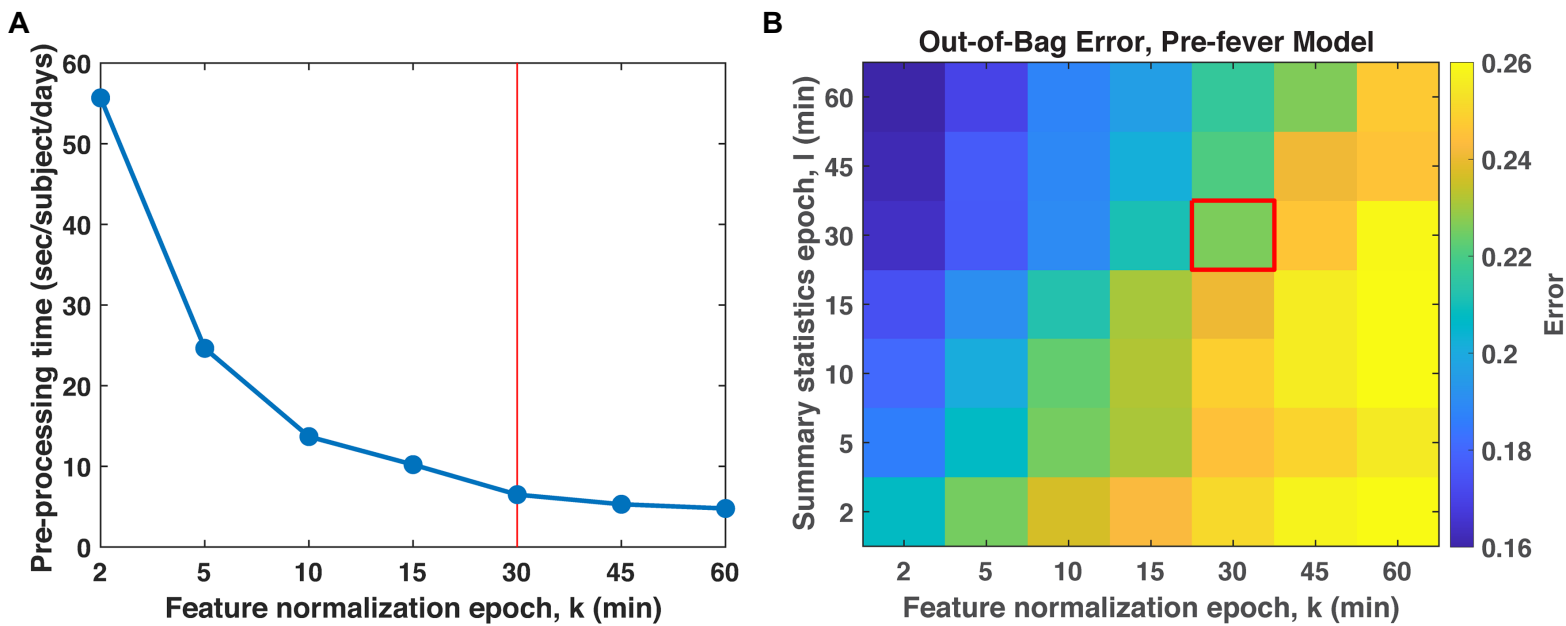
**(A)** Total pre-processing time per subject per day. **(B)** Classification accuracy as a function of the epoch lengths for feature normalization, *k*, and summary statistics, *l*. We chose *k* = *l* = 30 min (red box) as a compromise between these two cost objectives.

We begin by evaluating trade-offs for the *k*- and *l*-minute epoch length. Figure 3A shows the relative computation time for the data conditioning steps of section “Data Pre-Processing: Conditioning Physiological Data” as a function of the normalization epoch, *k*-minutes. Preprocessing was performed on a Dell desktop computer with dual Intel Xeon E5607 processors and 12GB RAM. Preprocessing is very time-consuming for the shortest epochs but the time burden decreases with increasing epoch length, leveling out around 30 min. Figure 3B shows the impact of both the feature normalization and summary statistics epoch lengths on classifier performance. In general, shorter epochs for the feature normalization are associated with lower errors and therefore better classification accuracy. In contrast, long epochs for the summary statistics provide better accuracy than short ones. The result suggests selecting *l* ≥ *k*, that is, the summary statistics epoch should be at least as long if not longer than the normalization interval. As a balance between processing time and classifier accuracy, we select our epochs as *k* = *l* = 30 min. With 48 epochs in a 24 h period and three summary statistics per physiological features listed in, we nominally compute 144 features per subject per day.

**Figure 4:**
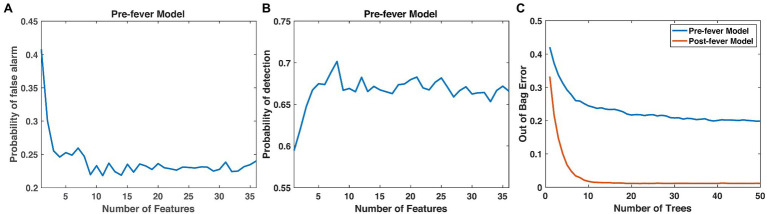
Justification for the number of features (10) and trees (15) in our random forest models. **(A)** *P_fa_* and **(B)** pre-fever *P_d_* plateau at around 10 features, and **(C)** the classification accuracy plateaus at around 15 trees.

Next, we optimize the random forest parameters, *n_trees_* and *n_features_*. As shown in Figure 4, both the false positive rate and the pre-fever Pd improve as *n_features_* increases from 1 to about 10, but performance plateaus beyond 10. Similarly, classifier accuracy improves as *n_trees_* increases but plateaus beyond about 15. We settle on a classifier composed of 15 trees grown on the 10 highest ranked features.

Table 4 shows the 10 highest-ranked features for each of the three partitions used in cross validation as well as the 10 highest-ranked features when we re-train the models on the full set of *N* = 20 animals. The ranked feature importance shows consistency with clinical symptomology, namely that core temperature-based features (mean and quantiles of temperature) in the post-fever model rank highest in importance. Before fever, however, ECG- and blood pressure-derived features are among the highest in feature importance, as has been reported at the earliest stages of sepsis (Korach et al., 2001; Chen and Kuo, 2007; Ahmad et al., 2009; Scheff et al., 2012). Among the blood pressure features, quantiles of systolic and diastolic aortic pressure rank as the most important. Among ECG-derived features, means and quantiles of QT intervals [corrected (Bazett, 1920; Fridericia, 2003) or not], RR intervals and PR intervals are routinely selected as those with the greatest predictive capability. Respiratory rate features derived from the intrathoracic pressure waveform were seldom ranked among the most important.

**Table 4:** Ranked importance of the 10 selected features for models trained with subjects from MARV IM, MARV aerosol and EBOV aerosol.

	Rank	3-fold cross-validation			All subjects (*N* = 20)
		Partition 1 (*N* = 6)	Partition 2 (*N* = 7)	Partition 3 (*N* = 7)	
Pre-fever	1	AOPDiastolic_Q25	PR_Mean	Temp_Mean	QRS_Mean
	2	AOPSystolic_Q75	RR_Q75	RR_Mean	Temp_Mean
	3	Temp_Mean	QT_Q25	PR_Mean	RR_Q75
	4	PR_Mean	Bazett_Q25	QT_Q75	AOPDiastolic_Q25
	5	Bazett_Mean	Temp_Mean	AOPSystolic_Q75	AOPAMean_Q25
	6	RR_Mean	QRS_Mean	PR_Q75	Bazett_Mean
	7	Temp_Q25	QRS_Q25	QT_Mean	QT_Q25
	8	Bazett_Q25	QRS_Q75	HR_Mean	PR_Q25
	9	Fridericia_Mean	PR_Q25	Bazett_Mean	AOPDiastolic_Mean
	10	AOPDiastolic_Mean	Temp_Q25	RespMean_Mean	QT_Mean
Post-fever	1	Temp_Mean	Temp_Mean	Temp_Mean	Temp_Mean
	2	PR_Mean	AOPSystolic_Mean	Temp_Q75	RR_Q75
	3	Temp_Q25	Temp_Q75	AOPDiastolic_Mean	AOPAMean_Mean
	4	AOPDiastolic_Q75	AOPSystolic_Q75	AOPSystolic_Q75	QRS_Mean
	5	Temp_Q75	Temp_Q25	RR_Q75	AOPDiastolic_Mean
	6	RespMean_Mean	AOPSystolic_Q25	Temp_Q25	Bazett_Q25
	7	RR_Mean	AOPAMean_Mean	AOPDiastolic_Q75	PR_Mean
	8	AOPDiastolic_Mean	QT_Q75	AOPDiastolic_Q25	AOPSystolic_Mean
	9	RespMean_Q75	HR_Q25	QT_Q75	Bazett_Mean
	10	RR_Q75	RR_Q75	HR_Mean	AOPDiastolic_Q25

**Figure 5:**
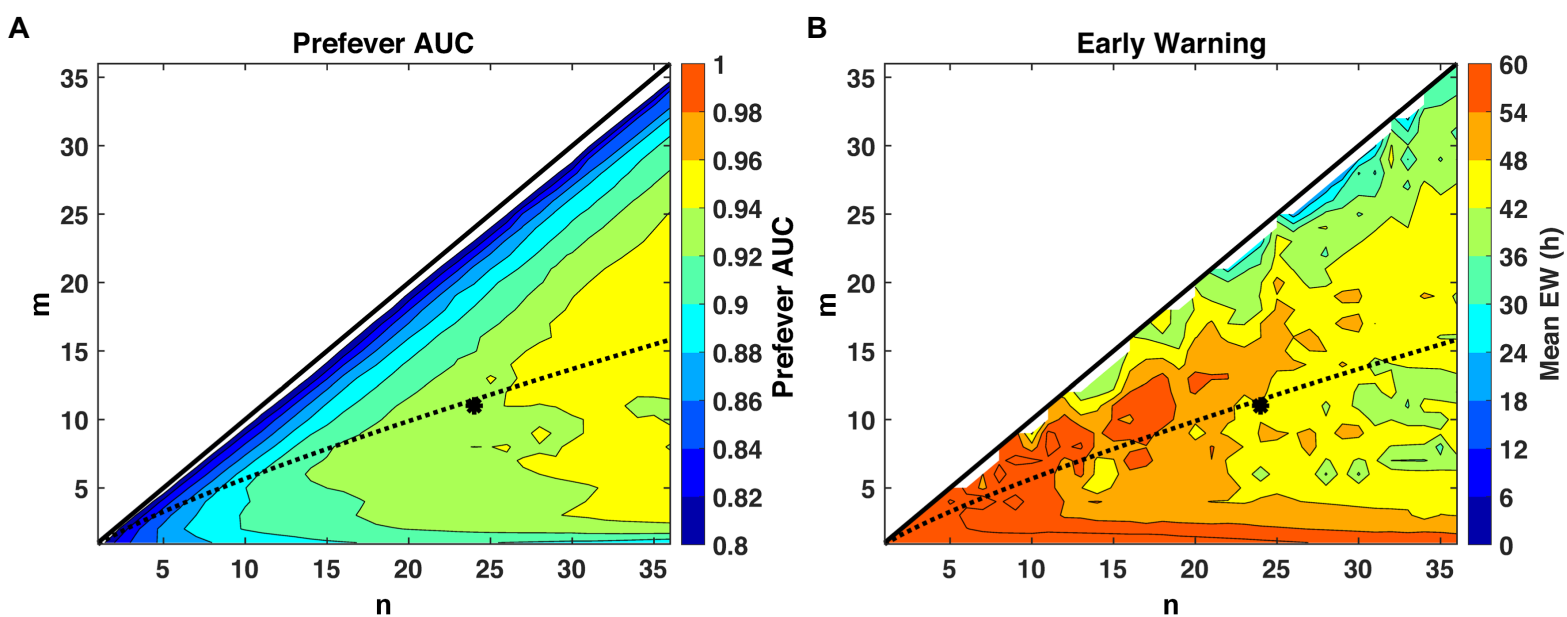
Performance evaluation across detection logic parameters m and n for a system *P_fa_* = 0.01. The theoretical optimal value (Shnidman, 1998) of m for a given n and Pfa is indicated by the dashed line, and our selected operating point of (*m* = 11, *n* = 24) is indicated by the asterisk. **(A)** AUC improves with larger values of n, while **(B)** small values of n promote earlier warning times (*Δt*) by limiting the buffer length required for a declaration decision.

The first stage threshold values, listed in Table 3, are estimated for the pre- and post-fever models to enforce a target *P_fa_* = 0.01. Using these first-stage thresholds, we see in Figure 5, that larger *n* (longer buffer length) enables slightly better detection capability in the sense of AUC, but at the expense of reduced early warning time. The estimated optimal threshold, denoted by the dashed line, is reasonably aligned with peak performance for both early warning time and AUC at each *n*, allowing for a methodical assignment of the second-stage threshold, *m_opt_*, given a binary integration window, *n*. In this analysis, we select *n* = 24 and *m* = *m_opt_* = 11 to achieve high AUC while maintaining a system latency of no more than 12 h.

### Detection Performance for 20 Subjects Exposed to Ebola or Marburg Virus

**Figure 6:**
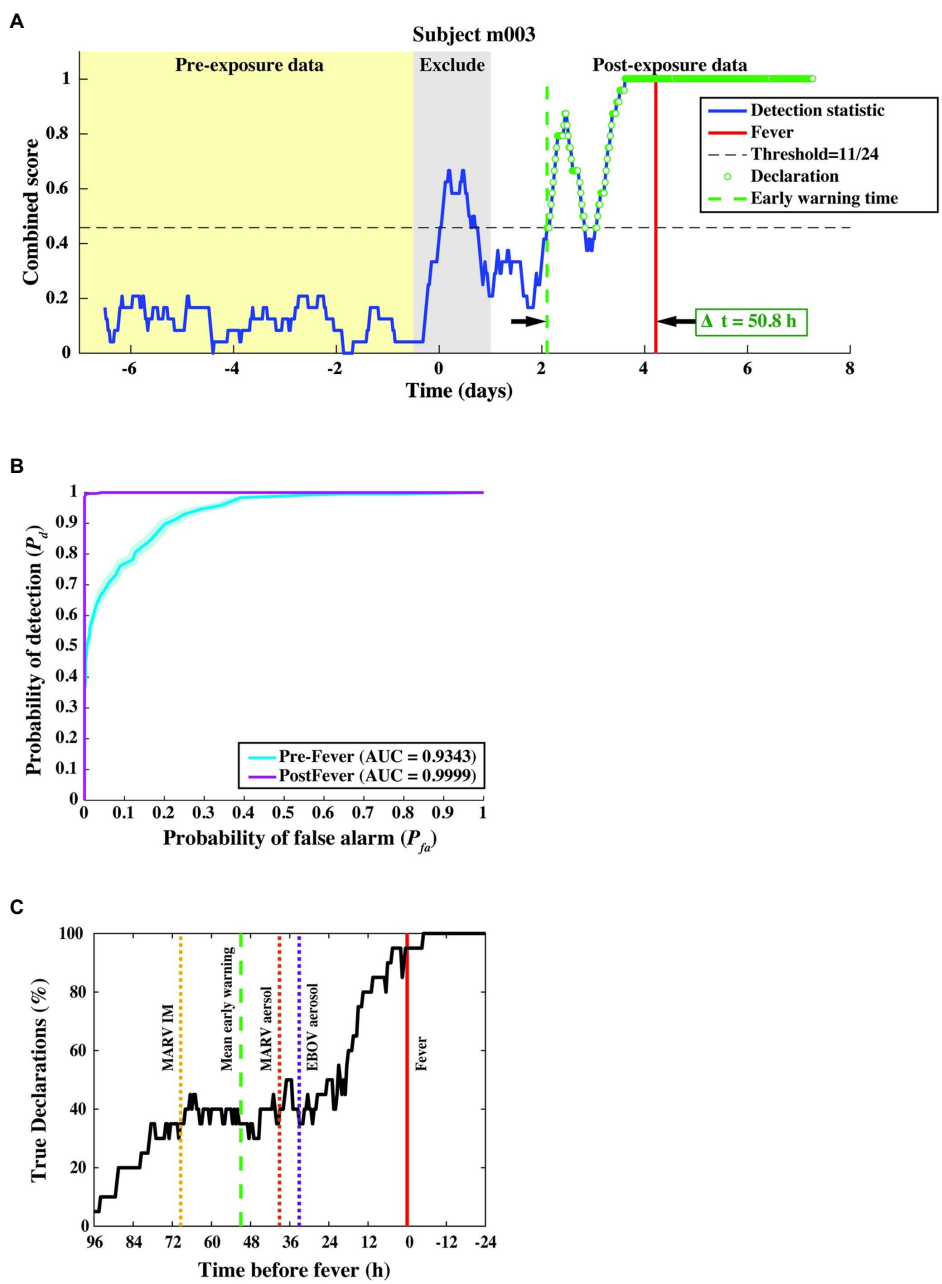
Algorithm output and performance measures from the three-fold cross-validation. **(A)** The combined score (blue curve) vs. time for a subject from the MARV aerosol exposure study, where samples declared as “exposed” are shown as green circles. The red vertical line indicates the fever onset time, and the green dashed vertical line denotes the first true positive declaration. The early warning time *Δt* is the interval between the green and red vertical lines. **(B)** ROC curve across 20 subjects, indicating nearly perfect performance after febrile symptoms and strong positive predictive power (AUC = 0.93 ± 0.01) before fever. **(C)** *P_d_* vs. time before fever. The mean Δ*t* for each of the three constituent studies is indicated by the dashed line. We find that half of the subjects are correctly identified as exposed at least 24–36 h before fever, regardless of the particular pathogen, exposure route, or target dose.

A time series of the combined score resulting from the two-stage detection process for a representative animal subject from the MARV aerosol study is shown in Figure 6A. The combined score, for this subject, remains below the detection threshold (dashed horizontal line) before virus challenge, rises sharply around exposure (which is excluded) due to anesthesia, then rises again at ~2 days post-exposure, where the first “exposed” declaration (dashed vertical green line) occurs.

In this cross-validation assessment, we evaluated performance over a total of 9,931 decision points from *N* = 20 subjects and found a system *P_d_* = 0.80 ± 0.01, a pre-fever *P_d_* = 0.56 ± 0.02, a system *P_fa_* = 0.01 ± 0.003, and a mean early warning time of *Δt_mean_* = 51 ± 12 h. Detailed performance metrics on each subject can be found in the Supplemental Material.

We further evaluated algorithm performance for all subjects with the family of ROC curves shown in Figure 6B, where the *P_d_* is separately evaluated against pre- and post-fever data samples. For this three-fold cross-validation, we find AUC = 0.93 ± 0.01 for pre-fever data, and AUC = 0.99 ± 0.001 for post-fever data.

Figure 6C shows a plot of correct declarations as a function of early warning time. This plot focuses on detectability in the pre-fever region for a threshold corresponding to *P_fa_* = 0.01. Mean early warning time, estimated for each pathogen exposure is shown as a dashed vertical line, which indicates individual differences between pathogens and exposure study conditions. Among these three studies, we see the earliest mean warning time for MARV IM exposure at *Δt_mean_* = 69 ± 16 h, while the two aerosol exposures, EBOV and MARV, have similar mean values at *Δt_mean_* = 33 ± 26 h and *Δt_mean_* = 39 ± 18 h, respectively.

### Applicability of Pre- and Post-fever Models to Other Pathogens

**Figure 7:**
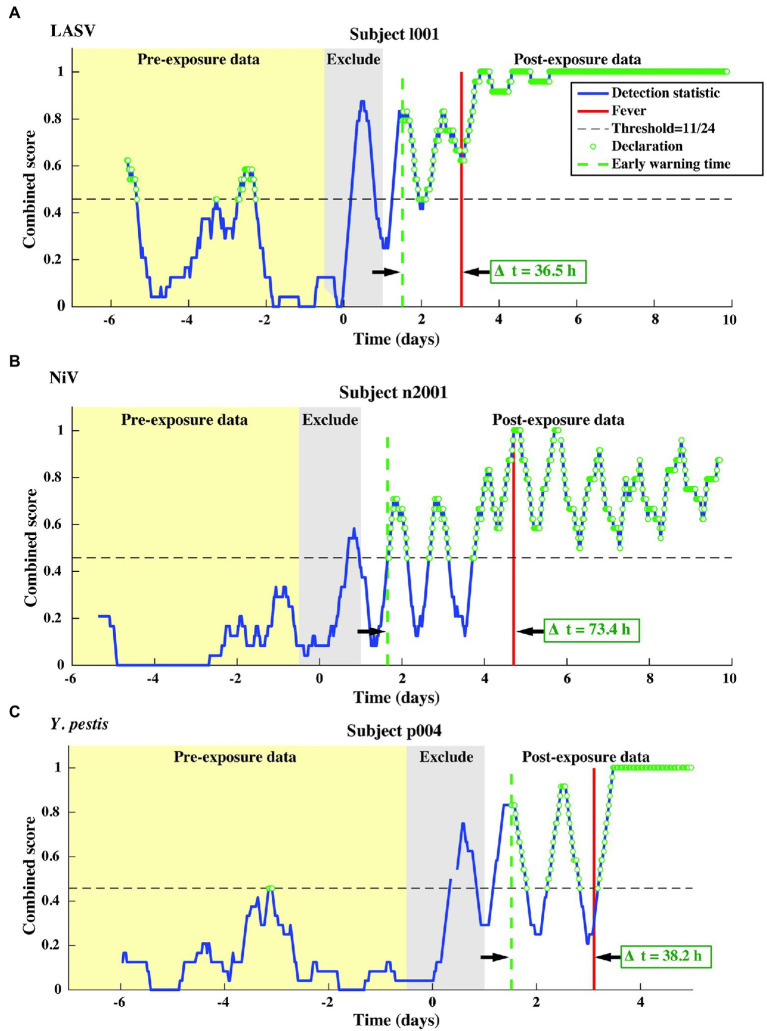
Representative single subject outputs from each of three independent datasets. Scores and declarations for: **(A)** LASV, **(B)** NiV, and **(C)** *Y. pestis*. Declarations made in the pre-exposure data represent false positives.

We test our pre- and post-fever models against data from the LASV aerosol, NiV intratracheal, and *Y. pestis* aerosol studies (Table 1, *N* = 13 subjects). The combined score vs. time is shown in Figure 7 for one representative subject for each pathogen.

**Figure 8:**
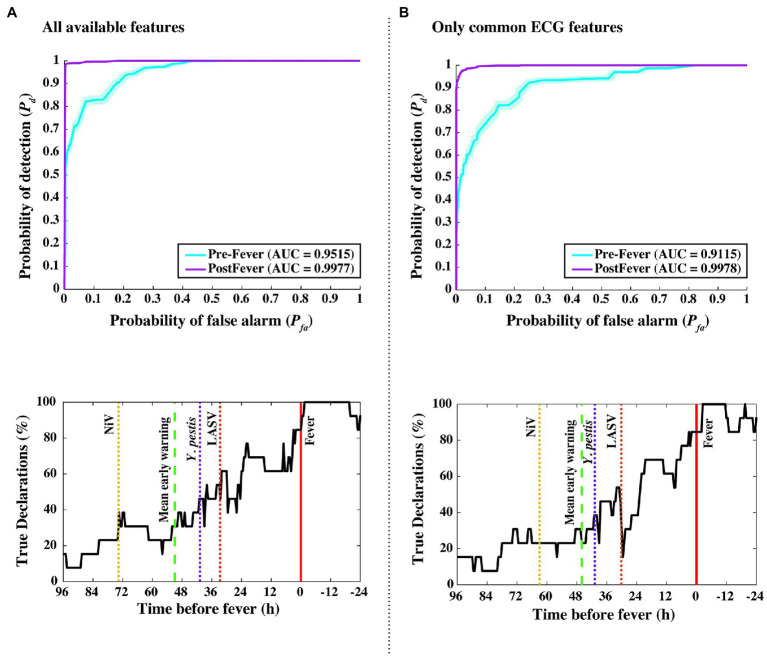
Performance measures from independent data set validations. ROC and detectability vs. time before fever curves were using **(A)** all available features from the implantable telemetry system (Table 2), and **(B)** using only features derived from the ECG waveform. Even when all temperature, blood pressure, and respiratory features are excluded, algorithm performance drops only slightly from Δ*t_mean_* = 51 to 46 h, and from pre-fever AUC = 0.95 to 0.91.

This independent validation set includes over 11,000 decision points including the supplemental baseline data from nine subjects that were otherwise excluded (the supplemental points contribute only toward *P_fa_*; they are excluded from *P_d_* calculations). The corresponding ROC curves and mean early warning times for the independent validation set are shown in Figure 8A. Again, detection performance against post-fever samples is nearly perfect, and we observe significant pre-fever positive predictive value of the model, with an AUC = 0.95 ± 0.01. Across the three pathogens, we find a system *P_d_* = 0.90 ± 0.007 and *P_fa_* = 0.03 ± 0.004, a pre-fever *P_d_* = 0.55 ± 0.03, and a mean early warning time of *Δt_mean_* = 51 ± 14 h. Calculating *Δt_mean_* for each pathogen exposure study individually, we find that the NiV IT study has the longest *Δt_mean_* = 75 ± 30 h (though NiV subjects also have the longest incubation period, ~5 days), and that LASV aerosol and *Y. pestis* aerosol exposure studies have *Δt_mean_* = 33 ± 26 h and *Δt_mean_* = 41 ± 25 h, respectively (with a mean incubation period ~3.5 days). A summary of the performance metrics from this independent validation data set are shown along with the cross-validation data set performance in Table 5.

**Table 5:** System performance metrics for the three validation conditions.

Primary objective	Training set	Test set	Mean *Δt* ± 95% CI (h)	Pre-fever AUC ± 95% CI	Post-fever AUC ± 95% CI	Pre-fever *P_d_* ± 95% CI	System *P_d_* and *P_fa_* ± 95% CI
Initial proof of concept	EBOV & MARV (Studies 1–3)	EBOV & MARV (Studies 1–3)	51 ± 12	0.93 ± 0.01	0.999 ± 0.001	0.56 ± 0.02	0.80 ± 0.010 0.01 ± 0.003
Extension to different pathogens	EBOV & MARV (Studies 1–3)	LASV, NiV & *Y. pestis* (Studies 4–6)	51 ± 14	0.95 ± 0.01	0.998 ± 0.001	0.60 ± 0.03	0.90 ± 0.007 0.03 ± 0.004
Feasibility for wearable devices	EBOV & MARV (Studies 1–3) Only ECG-derived features	LASV, NiV & *Y. pestis* (Studies 4–6) Only ECG-derived features	46 ± 14	0.91 ± 0.001	0.998 ± 0.001	0.55 ± 0.03	0.89 ± 0.008 0.03 ± 0.004

### Emulation of Early Warning Performance for Wearable Systems

As an *in silico* simulation for degrading our animal dataset to what may be collected using a wearable monitoring device for humans, we reduced the considered feature set to include only ECG-derived features such as RR, QT, QRS, and PR intervals. Figure 8 compares our algorithm performance using all available features (Figure 8A) and features derived only from the ECG waveform (Figure 8B). For the degraded feature set, we see only modest decreases in performance with *Δt_mean_* = 46 ± 14 h, pre-fever *P_d_* = 0.55 ± 0.03, and system *P_d_* = 0.89 ± 0.008 and *P_fa_* = 0.03 ± 0.004.

## Discussion

Non-biochemical detection of pathogen incubation periods using only physiological data presents an enabling new tool in infectious disease care. Previous work has shown that reducing transmission during the viral incubation period is as or more effective an intervention as reducing the inherent transmissibility (*R_0_*) of the pathogen in controlling emerging outbreaks (Fraser et al., 2004). Over the past year, during the COVID-19 pandemic, a number of efforts have reported results for detecting COVID-19 using wearable devices such as smart watches and smart rings. In these observational studies with human subjects, reported AUCs range from 0.69 (Quer et al., 2021) to 0.77 (Natarajan et al., 2020) including data from the symptomatic period while the reported probability of detection for data restricted to the pre-symptomatic period was around *P_d_* = 0.20 at *P_fa_* = 0.05 (Miller et al., 2020; Natarajan et al., 2020). In our effort, which leverages animal model studies, we had three primary objectives. *First*, we aimed to understand the upper limits of detecting illness during the asymptomatic incubation period using medical grade devices under controlled conditions. *Second*, we sought to determine whether the detection was specific to a particular pathogen. And *third*, we investigated the feasibility of extending the detection capability to wearable devices under controlled conditions.

We developed detection algorithms composed of random forest classifiers coupled with novel declaration logic to provide early warning of illness using physiological waveforms collected from non-human primates infected with several pathogens. We then evaluated the detection capability of our algorithms under three distinct scenarios.

First, to answer the fundamental question of how well pathogen exposure can be detected based on physiological measurements, we evaluated data from the subset of *N* = 20 animal subjects from the EBOV aerosol, MARV aerosol, and MARV IM studies using a 3-fold cross-validation approach. In this evaluation, we achieved a pre-fever detection performance of *P_d_* = 0.56 ± 0.02 with *P_fa_* = 0.01 ± 0.004 and a mean early warning time of *Δt_mean_* = 51 ± 12 h. Second, to determine whether this capability is specific to the pathogen, we took a model trained on the EBOV and MARV studies and applied it to an independent dataset of *N* = 13 animal subjects from the LASV, NiV, and *Y. pestis* studies. Evaluating this independent dataset, we found very comparable performance with pre-fever detection of *P_d_* = 0.55 ± 0.03 with *P_fa_* = 0.03 ± 0.004 and mean early warning time of *Δt_mean_* = 51 ± 14 h. This successful extension for a hemorrhagic fever virus (LASV), a henipavirus (NiV), and a gram-negative coccobacillus (*Y. pestis*) suggests algorithm insensitivity to particular pathogens, and possible generalization for novel or emerging agents for which data has not or cannot be collected. Third, we emulated a scenario for a non-invasive wearable device by restricting the classifier to use only ECG-derived features such as RR, QT, QRS, and PR intervals. Again, performance was comparable, with only a slight decrease in mean early warning time: pre-fever *P_d_* = 0.55 ± 0.03 with *P_fa_* = 0.03 ± 0.004 and *Δt_mean_* = 46 ± 14 h. These results were achieved in the absence of core temperature, and hence without direct observation of febrile symptoms. Performance from this ECG-only feature set suggests that the implementation of this approach is possible with non-invasive wearable devices.

During the non-symptomatic pre-fever stage of infection, where early warning is most meaningful, we observed strong positive predictive value with ECG and temperature-related features emerging as the most important features. In the febrile prodrome stage of infection, core temperature-derived features were consistently ranked most important. We also observed differences in the mean early warning time based on the route of exposure (intramuscular vs. intratracheal vs. aerosol) and pathogen. The NiV IT and MARV IM studies, which used exposure routes that allow for more precise control of dose, had the longest early warning at *Δt_mean_* = 75 ± 30 h and *Δt_mean_* = 69 ± 16 h, respectively. Across the aerosol exposures, mean early time was considerably lower with *Δt_mean_* = 41 ± 25 h for *Y. pestis*, *Δt_mean_* = 39 ± 18 h for MARV aerosol, *Δt_mean_* = 33 ± 26 h for EBOV, and *Δt_mean_* = 33 ± 26 h for LASV. These differences potentially highlight a dose–response associated with the route of exposure and the mean early warning time of the physiological perturbations.

We postulate that underlying immuno-biological events of the innate immune system are responsible for the observable changes in the physiological signals that enable this early warning capability. In particular, the systemic release of pro-inflammatory chemokines and cytokines from infected phagocytes (Hayden et al., 1998; Leroy et al., 2000; Gupta et al., 2001; Hensley et al., 2002; Martinez et al., 2008; Connor et al., 2015), as well as afferent signaling to the central nervous system (Tracey, 2002; Beishuizen and Thijs, 2003), are recapitulated in hemodynamic, thermoregulatory, or cardiac signals. For instance, prostaglandins (PG) are upregulated upon infection [including EBOV (Geisbert et al., 2003; Wahl-Jensen et al., 2011)] and intricately involved in the non-specific “sickness syndrome” (Saper et al., 2012); the PGs are also known to be potent vascular mediators (Funk, 2001) and endogenous pyrogens (Sugimoto et al., 2000; Ek et al., 2001). Recent work has shown how phagocytic immune cells directly modulate electrical activity of the heart (Hulsmans et al., 2017). Past work has clarified how tightly integrated, complex, and oscillating biological systems can become uncoupled (Godin and Buchman, 1996; Goldberger et al., 2002; Bravi et al., 2011) during trauma (Cancio et al., 2013) or critical illness (Scheff et al., 2012, 2013a), which would be captured in the comprehensive, multi-modal physiological datasets used in our present work.

Our study has several key strengths. *First*, using non-human primate data collected under extremely controlled environments, we are able to set the bar for the upper limits of early warning detections, showcasing that recent efforts for early warning of COVID-19 using wearables have potential for significant improvement. *Second*, we show that the body’s immunological response is not specific to the pathogen. This result is of great importance as algorithms developed for COVID-19 can likely provide early warning for influenza and other illnesses, providing a tool that can be used to steer public health policies and individual medical care. *Third*, to the best of our knowledge, we are the first to highlight the potential relation between early warning time and the route of pathogen exposure. *Fourth*, the importance of ECG features in detecting an immunological response to pathogen provides impetus for device manufacturers to leverage wearables as important tools for personal and public health.

We also note some limitations of our study. First, our sample size for the animal studies is relatively small. While we compensate for the small *N* by employing a case cross-over methodology, where in each subject is a control for themselves, we believe our results can be strengthened with a larger *N*. Second, while *Δt* for an individual subject is very useful clinically, we note that for our datasets the *mean* early warning time is potentially unstable due to the low sample sizes. Third, in an operational, clinically useful early warning system, it may be desirable to calculate *P_d_* and *P_fa_* on a per-device, per-subject, or per-day basis. However, given our sample size, we calculated *P_d_* and *P_fa_* across all 30-min epochs. This approach penalizes for false negatives (missed detections) that may occur after an initial early warning declaration is made, and thus provides a conservative estimate of sensitivity on a per-subject basis. Furthermore, we chose a target system *P_fa_*~0.01 based on the limited sample size, but this could lead to an unacceptable daily false alarm rate of about one declaration every 2 days (for 30 min epochs). We estimate *P_fa_* should be ~10^−3^ or less, which corresponds to one false alarm approximately every 3 weeks of continuous monitoring (again, for 30 min epochs). Reducing this critical system parameter to more clinically acceptable levels is the subject of on-going work, and will require larger sample sizes or more refined processing algorithms. Finally, the effect of physiological confounders, such as intense exercise, arrhythmias, lifestyle diseases, and autochthonous or annual infections, has not been explored in this initial study.

## Conclusion

Detecting pathogen exposure before symptoms are self-reported or overtly apparent affords great opportunities in clinical care, field uses, and public health measures. However, given the consequences of using some of these interventions and the lack of etiological agent specificity in our algorithm, we envision this current approach (after appropriate human testing) to be a trigger for “low-regret” actions rather than necessarily guiding medical care. For instance, using our high sensitivity approach as an alert for limited high specificity confirmatory diagnostics, such as sequencing or PCR-based, could lead to considerable cost savings (an “alert-confirm” system). Public health response following a bioterrorism incident could also benefit from triaging those exposed from the “worried well.” Ongoing work focuses on adding enough causative agent specificity to discern between bacterial and viral pathogens; even this binary classification would be of use for front-line therapeutic or mass casualty uses. Eventually, we envision a system that could give real-time prognostic information, even before obvious illness, guiding patients, and clinicians in diagnostic or therapeutic use with better time resolution than ever before.

## Data Availability Statement

Preprocessed physiological data for the animal studies considered here are provided in the article/Supplementary Material.

## Ethics Statement

The animal study was reviewed and approved by US Army Medical Research Institute of Infectious Diseases (USAMRIID) Institutional Animal Care and Use Committee (IACUC).

## Author Contributions

AS, GC, CC, AH, and WP conceptualized the study. LM, SD, TP, and MH curated the data. LM, SD, TP, MH, and SS contributed to formal data analysis and visualization. LM, SD, GC, JF, and AR developed the methodology. LH, AG, JT, SJ, BP, FR, AH, and WP were involved in the animal research, data acquisition, and/or data interpretation. AS was the principal investigator. AS, LM, SD, and KC wrote the manuscript. LM and SD contributed equally to this work. The manuscript was written and all work was done prior to GC joining Amazon. All authors contributed to the article and approved the submitted version.

## Author Disclaimer

Any opinions, findings, conclusions, or recommendations expressed in this material are those of the authors and do not necessarily reflect the views of the United States Government or reflect the views or policies of the US Department of Health and Human Services.

## Conflict of Interest

The authors declare the following competing financial interests: patent US 10332638B2 was issued June 2019 and provisional US patent application 62/337,964 was filed May 2016.

## Publisher’s Note

All claims expressed in this article are solely those of the authors and do not necessarily represent those of their affiliated organizations, or those of the publisher, the editors and the reviewers. Any product that may be evaluated in this article, or claim that may be made by its manufacturer, is not guaranteed or endorsed by the publisher.
