# Supplementary material for: Detecting Pathogen Exposure During the Non-symptomatic Incubation Period Using Physiological Data: Proof of Concept in Non-human Primates
Source: Front Physiol. 2021 Sep 3;12:691074. doi: 10.3389/fphys.2021.691074 (PMC8451540; doi:10.3389/fphys.2021.691074)
Supplement: Supplementary file 1 [file Data_Sheet_1.docx]

Supplementary Material

# Comparison of Classification Methods

We experimented with other supervised classification methods in addition to random forests, including naïve Bayes, and k-nearest neighbors, but chose random forest classifiers for several reasons, foremost being that random forests make no assumptions of statistical independence of features, which is useful given highly correlated physiological feature sets.

Figure S1 shows the cross-validation performance on the MARV and EBOV studies following the 2-stage detection as described in the Methods Section. The top row shows the nominal algorithm performance using random forests. For the next two rows, the random forests were replaced with two other classifiers, naïve Bayes and k-nearest neighbor, while all of the remaining detection logic is held constant. Of the three classifiers, random forests gave the best performance in terms of both AUC and mean early warning time. Though many other classifiers could be considered, the purpose of this study is to prove the concept of early detection rather than to exhaustively evaluate learning methods; improved classification approaches are the subject of on-going work.

# Detailed Detection Performance

**Table S1** provides detection details at the per-subject level for the three performance evaluations presented. The Study column corresponds to the non-human primate model studies summarized in Table 2. In the cross-validation study, the *N*=20 animal subjects of the MARV and EBOV studies are divided into three partitions as listed in the table and used for both training and testing. For the independent validation study, training and testing are performed using animal subjects from different studies, as described. The final rows detail model performance using only ECG-derived features in the independent dataset validations. False Declarations refers to the number of false alarms in the baseline period, Data Samples (baseline class) is the number of data samples from the baseline period, True Declarations is the number of correctly detected post-exposure samples, and Data Samples (exposed class) is the number of data samples from the post-exposure period (including pre- and post-fever).

|  | ROC curve | Probability of Detection vs Time |
| --- | --- | --- |
| Random Forest | 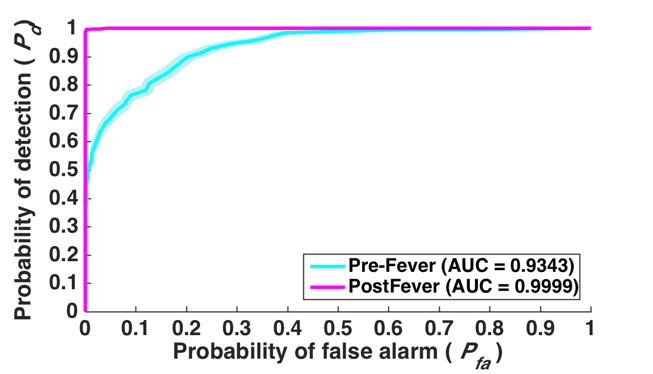 | 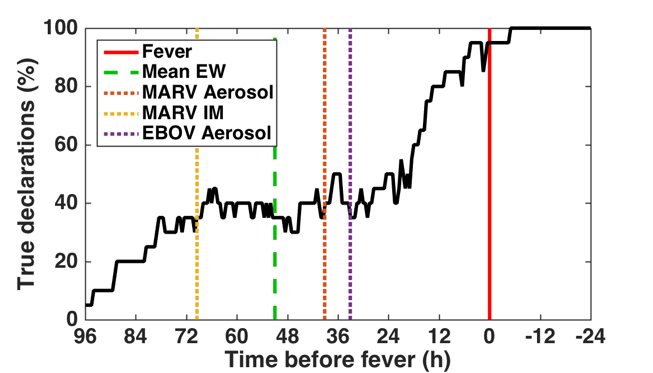 |
| Naive Bayes | 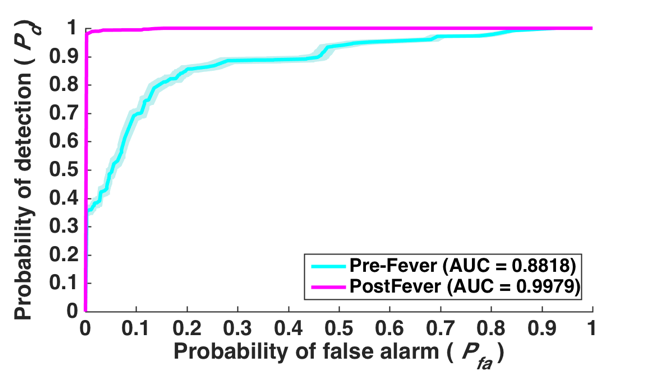 | 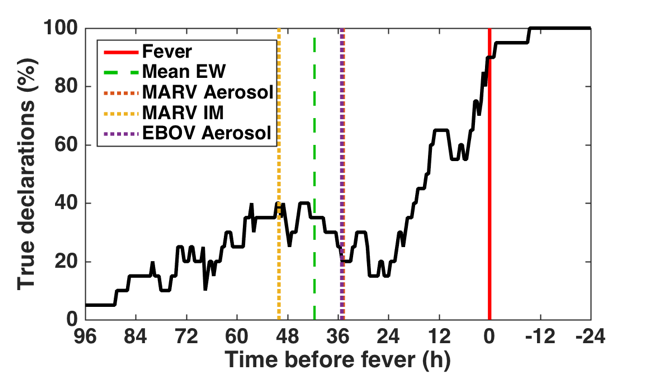 |
| kNN | 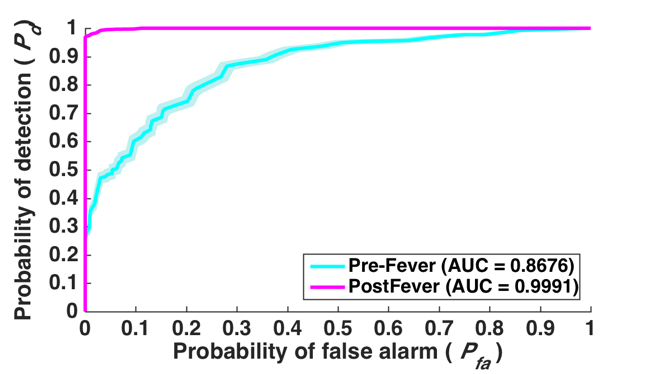 | 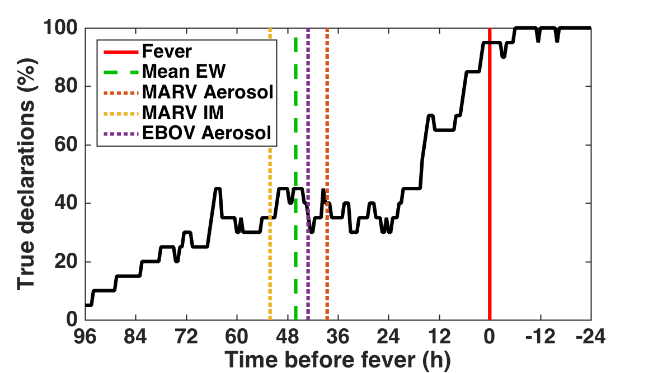 |

Figure S1: ROC curves showing performance comparison of three different classifiers. Random Forests, Naïve Bayes, and k-Nearest Neighbors methods were compared using the three-fold cross validation dataset. Shading around the ROC curves indicates 95% confidence intervals.

Table S1: Declaration performance for each subject in all validation experiments.

| **Training Set** | **Test Set** | **Study** | **Subject** | **Early Warning Time**  **(***Δt,* **hours)** | **False**  **Declarations** | **Data Samples  (Baseline class)** | **True**  **Declarations** | **Data Samples (Exposed class)** |
| --- | --- | --- | --- | --- | --- | --- | --- | --- |
| EBOV and MARV Studies  (3 partitions for training,  tuning parameters, performance validation) | Partition 1 | 3 | e1001 | 50.3 | 12 | 273 | 192 | 213 |
|  | Partition 1 | 3 | e1004 | 10.3 | 0 | 281 | 129 | 232 |
|  | Partition 3 | 3 | e1005 | 65.7 | 0 | 279 | 176 | 253 |
|  | Partition 2 | 3 | e1009 | 51.5 | 0 | 279 | 207 | 283 |
|  | Partition 3 | 3 | e1011 | 14.9 | 1 | 279 | 120 | 189 |
|  | Partition 2 | 3 | e1015 | 5.8 | 0 | 279 | 161 | 258 |
|  | Partition 1 | 1 | mra001 | 37.1 | 0 | 289 | 232 | 297 |
|  | Partition 3 | 1 | mra002 | 14.4 | 0 | 289 | 225 | 297 |
|  | Partition 2 | 1 | mra003 | 50.8 | 0 | 289 | 240 | 277 |
|  | Partition 3 | 1 | mra004 | 31.1 | 40 | 289 | 216 | 280 |
|  | Partition 2 | 1 | mra005 | 62.4 | 0 | 289 | 246 | 259 |
|  | Partition 1 | 2 | mci001 | 76.8 | 0 | 108 | 259 | 305 |
|  | Partition 1 | 2 | mci002 | 65.7 | 0 | 108 | 222 | 315 |
|  | Partition 2 | 2 | mci003 | 82.0 | 0 | 106 | 292 | 375 |
|  | Partition 3 | 2 | mci004 | 76.1 | 0 | 107 | 367 | 367 |
|  | Partition 3 | 2 | mci005 | 32.3 | 0 | 107 | 199 | 331 |
|  | Partition 2 | 2 | mci006 | 87.0 | 0 | 108 | 339 | 359 |
|  | Partition 3 | 2 | mci007 | 73.8 | 0 | 107 | 355 | 355 |
|  | Partition 2 | 2 | mci008 | 65.1 | 0 | 107 | 256 | 303 |
|  | Partition 1 | 2 | mci011 | 66.2 | 0 | 107 | 267 | 303 |
| EBOV and MARV Studies  (N=20 subjects) | LASV | 4 | l001 | 36.5 | 38 | 245 | 400 | 402 |
|  | LASV | 4 | l002 | 61 | 0 | 210 | 540 | 540 |
|  | LASV | 4 | l003 | -0.9 | 0 | 247 | 305 | 398 |
|  | LASV | 4 | l004 | 33.9 | 0 | 242 | 1776 | 1891 |
|  | NiV | 5 | n1001 | 97.5 | 0 | 232 | 686 | 848 |
|  | NiV | 5 | n1002 | 81.4 | 17 | 232 | 162 | 282 |
|  | NiV | 5 | n1003 | 22.9 | 0 | 232 | 355 | 362 |
|  | NiV | 5 | n1004 | 93.5 | 16 | 232 | 828 | 848 |
|  | NiV | 5 | n2001 | 73.4 | 0 | 234 | 335 | 393 |
|  | *y. pestis* | 6 | p001 | 20 | 22 | 261 | 81 | 88 |
|  | *y. pestis* | 6 | p002 | 76.2 | 0 | 263 | 192 | 206 |
|  | *y. pestis* | 6 | p003 | 28.8 | 2 | 262 | 97 | 98 |
|  | *y. pestis* | 6 | p004 | 38.2 | 4 | 260 | 131 | 165 |
| EBOV and MARV Studies  (N=20 subjects)  restricted to ECG-derived features | LASV | 4 | l001 | 36.5 | 6 | 245 | 375 | 402 |
|  | LASV | 4 | l002 | 61.0 | 0 | 210 | 540 | 540 |
|  | LASV | 4 | l003 | -2.9 | 5 | 247 | 288 | 398 |
|  | LASV | 4 | l004 | 25.9 | 0 | 242 | 1810 | 1891 |
|  | NiV | 5 | n1001 | 97.5 | 20 | 232 | 688 | 848 |
|  | NiV | 5 | n1002 | 79.4 | 5 | 232 | 166 | 282 |
|  | NiV | 5 | n1003 | 21.9 | 11 | 232 | 353 | 362 |
|  | NiV | 5 | n1004 | 93.5 | 31 | 232 | 833 | 848 |
|  | NiV | 5 | n2001 | 22.4 | 15 | 234 | 270 | 393 |
|  | *y. pestis* | 6 | p001 | 20.0 | 0 | 261 | 65 | 88 |
|  | *y. pestis* | 6 | p002 | 76.2 | 2 | 263 | 183 | 206 |
|  | *y. pestis* | 6 | p003 | 28.8 | 0 | 262 | 98 | 98 |
|  | *y. pestis* | 6 | p004 | 38.2 | 0 | 260 | 138 | 165 |
